# Optimization of protein electrophoresis test orders: results after the implementation of a protocol based on scientific recommendations

**DOI:** 10.1515/almed-2025-0129

**Published:** 2026-05-18

**Authors:** Manuel Baladrón-Segura, Nuria Alonso-Castillejos, María Lorena Navas-Gómez, Sara Martín-Gabriel, Carmen González-Sama, Sofía Cabezas-Marcos, Marta Capilla-Díez, Beatriz Calvo-Antón

**Affiliations:** Department of Clinical Biochemistry, Río Hortega University Hospital, Valladolid, Spain; Hospital Pharmacy Department, Hospital Clínico Universitario of Valladolid, Valladolid, Spain

**Keywords:** demand management, diagnostic test optimization, monoclonal gammopathy, quality indicators, serum protein electrophoresis

## Abstract

**Objectives:**

Serum protein electrophoresis is a valuable tool for diagnosing and monitoring monoclonal gammopathies, but its widespread use outside this context may be clinically unjustified. To promote more rational test ordering, a demand optimization protocol has been designed and implemented in the Valladolid Oeste Health Area (ASVAO), aligned with AEBM-ML and SEQC-ML recommendations.

**Methods:**

A retrospective observational and interventional study was conducted. The protocol included automated request blocks for patients under 40, revision of analytical profiles, electronic alerts, and automatic inclusion based on specific biochemical alterations. The protocol was disseminated and training was provided to ordering physicians in both primary care and hospital settings. Structural, process, and activity indicators have been monitored from January 2023 to June 2025.

**Results:**

Orders in patients under 50 years decreased from 23.5 % in 2023 to 14.1 % in 2025. The proportion of orders for patients ≥50 years exceeded the target (>0.80) in 2025. Inappropriate analytical profiles have been eliminated, although the rate of orders without clinical or analytical justification remained elevated (0.53 in 2025). Test volume per inhabitant remained stable. Primary Care, Gastroenterology, Nephrology, and Pediatrics significantly reduced testing in younger patients, while Rheumatology increased it.

**Conclusions:**

The protocol effectively improved test appropriateness, particularly regarding age targeting and the removal of automated orders. However, unjustified orders persist. Ongoing monitoring of indicators is recommended to consolidate improvements and address deviations.

## Introduction

Serum electrophoresis proteinogram (SPEP) is a widely used diagnostic tool in laboratory medicine to detect and monitor monoclonal gammopathies. However, the indiscriminate use of this tool when it is not clinically indicated may result in overdiagnosis, cause unnecessary anxiety to patients, increase healthcare expense, and overburden laboratory departments, without a clear clinical benefit [1], 2].

Aware of this situation, scientific societies, including the Spanish Association of Medical Biopathology-Laboratory Medicine (AEBM-ML) and the Spanish Society of Laboratory Medicine (SEQC-ML), in line with the international *movement “Choosing Wisely”*, have issued explicit recommendations aimed at restricting the use of SPEP to cases with clinical suspicion of monocolonal gammopathy or suggestive biochemical findings.

In 2024, the Spanish Society of Laboratory Medicine (SEMEDLAB) was established through the merger of the AEBM-ML, the Spanish Association of Clinical Laboratory (AEFA), and the SEQC-ML. In this paper, the former acronyms will be used to cite documents issued previously to the merger, whereas SEMEDLAB will be used for more recent documents [3], [4], [5].

In line with these recommendations and within the framework of the national project “Compromiso por la Calidad de las Sociedades Científicas en España” *(“Scientific Societies Committed to Quality in Spain”)* promoted by the Spanish Ministry of Health, a protocol was designed to optimize SPEP orders in the Valladolid Oeste Health District (ASVAO). The Protocol – approved in June 2024 – was aimed at optimizing SPEP use through a set of clinical and laboratory criteria and by defining follow-up indicators to assess its impact over time [4], 5].

There is cumulative evidence that monoclonal gammopathies are most frequently diagnosed in patients older than 50. The estimated incidence of this condition is 45–60 cases per million people, with an average age at diagnosis of 65 years. Accordingly, routine testing in younger patients lacks clinical justification, in the absence of consistent signs or symptoms such as anemia of unknown origin, hypercalcemia, kidney failure, or bone lesions [6], [7], [8].

This study describes the process and methods adopted to develop and implement the protocol and summarizes the evaluation conducted to assess the effectiveness of the protocol in promoting a rational use of SPEPs.

Recommendations aimed at optimizing SPEP demand according to the leading clinical laboratory scientific societies [3], 9], 10]:

– In patients younger than 50 years with no clinical suspicion of monoclonal gammopathy, SPEP is not recommended (AEBM-ML/SEQC-ML).
– In the pediatric population, SPEP testing is not indicated (SEQC-ML).
– General population screening through SPEP testing is not recommended (SEQC-ML).
– In the event of suspected monoclonal gammopathy (e.g. unexplained anemia, bone lesions, kidney failure hypercalcemia, among other signs), the SPEP test is indicated (AEBM-ML/SEQC-ML).
– SPEP should not be repeated within 12 months in patients without a diagnosis of monoclonal gammopathy and without new clinically relevant findings (SEQC-ML).
– To screen for isolated proteins in serum, targeted quantification methods should be used instead of SPEP (SEQC-ML/AEBM-ML).
– In the presence of biochemical abnormalities suggestive of gammopathy – even though SPEP is not included in the request – it is recommended that the laboratory includes the SPEP test.
– The unjustified inclusion of SPEP in laboratory profiles should be reviewed and eliminated in the absence of a clear clinical indication (AEBM-ML/SEQC-ML).

## Materials and methods

### Study design

A retrospective, observational, intervention study was carried out to evaluate the effectiveness of a SPEP request optimization protocol. The scope of the study includes primary and secondary care centers located in the Valladolid Oeste Health District (ASVAO). The study focuses on elective demand for SPEP tests.

**Figure 1:**
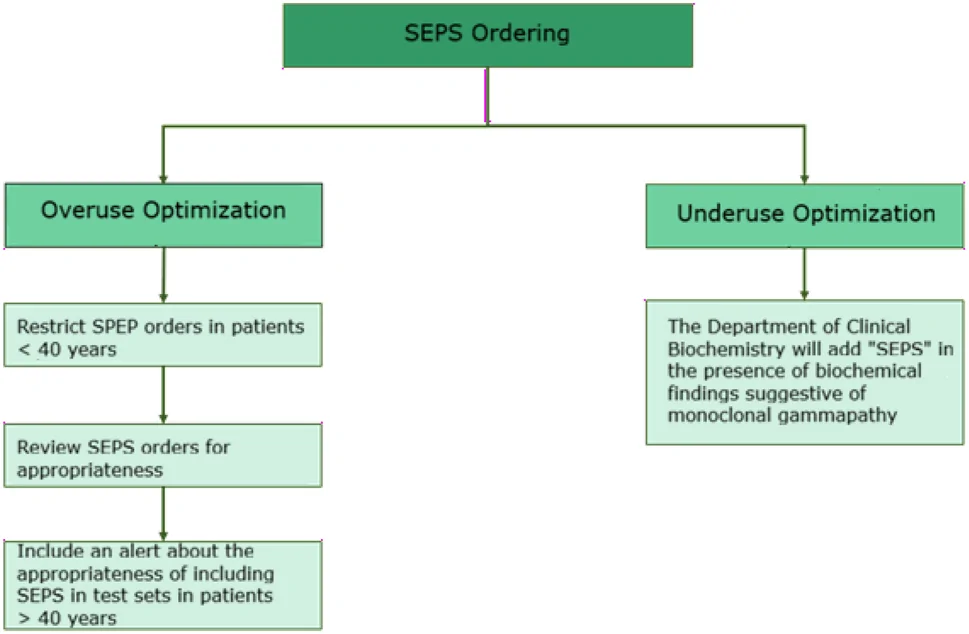
Algorithm for assessing the appropriateness of SEPS order practices.

Laboratory activity data was extracted from the Laboratory Information System (LIS) MODULAB^®^. An initial search for SPEP tests was performed between January 1 and December 31, 2023 to evaluate the baseline situation and identify potential deviations in SPEP request practices (Figure 1). Evaluation of the protocol’s implementation was performed by comparing data from two periods: from January 1 to December 31, 2024 – corresponding to the implementation phase – and from January 1 to June 30, 2025, during which the protocol was fully operational.

A 50-year epidemiological cutoff was used in descriptive analyses to enable comparison with the literature, whereas the 40-year operational cutoff for patients remained in place throughout the intervention.

### Protocol development process

In accordance with AEBM-ML and SEQC-ML recommendations, the protocol was developed by a multidisciplinary team including professionals from the Department of Clinical Biochemistry, the Department of Hematology, and Primary Care. The Protocol was approved by the Ethics Committee of ASVAO on June 27, 2024.

The interventions proposed included:

(1) **Optimizing unnecessary SPEP orders:**
  – Automatically restricting PSEP o~rders in patients younger than 40. In case the laboratory receives a justification for the request, the restriction is removed.
    In agreement with the Department of Hematology, the minimum age for PSEP referral was established at 40 years. The literature occasionally defines age threshold at 50 years; however, we sought to optimize specificity for the younger population, reduce unnecessary orders and prioritize resources.
    To this end, a thorough analysis of the 40–49 age range was conducted, involving an individual evaluation based on clinical and laboratory criteria [9].
  – Reviewing laboratory profiles that routinely include the SPEP test.
  – Including an electronic notification in the hospital’s laboratory order entry system ordering evidence supporting clinical suspicion of monoclonal gammopathy.
(2) **Optimizing the appropriateness of SPEP orders:**
  – Including PSEP tests automatically in patients with suggestive biochemical findings, even though the test had not been expressly included.
  – Adopting the following cut-off values to include PSEP in laboratory test orders:
    – Anemia: hemoglobin below the lower limit of normality. Reference values in women: 12–16 g/dL; in men: 12–18 g/dL.
    – Calcium (serum): above the upper limit of normality. Reference values 8.8–10.6 mg/dL.
    – Total serum proteins: above the upper limit of normality. Reference values: 6.6–8.3 g/dL.
    – Kidney function impairment: abnormal creatinine and/or estimated glomerular filtration rate (CKD-IPE 2009) with respect to previous studies. Reference values for creatinine by the compensated Jaffe method: 0.6–1.2 mg/dL; Estimated glomerular filtration ranges: G1: normal or elevated (>90 mL/min/1.73m^2^); G2: mildly reduced (60–89 mL/min/1.73m^2^); G3a: a moderately reduced (45–59 mL/min/1.73m^2^); G3b: moderately-to-severely reduced (30–44 mL/min/1.73m^2^); G4: severely reduced (15–29 mL/min/1.73m^2^) and G5: kidney failure (<15 mL/min/1.73m^2^).

Other relevant clinical and biochemical parameters for PSEP indication include classic immunoparesis, nephrotic syndrome, or significantly elevated protein levels, to name a few.

### Protocol distribution and training for ordering physicians

The protocol was distributed to all Hospital departments via the organizational e-mail.

A total of 12 clinical sessions were held both in Primary Care (eight PC centers in the Valladolid Oeste Health District (ASVAO)) and specialty care units including the Units of Clinical Laboratory Chemistry, Gastroenterology, Hematology, and Nephrology at Río Hortega University Hospital in Valladolid.

The purpose of these sessions was to define a battery of measures and provide training to ordering physicians to optimize PSEP demand.

### Evaluation indicators

A total of four indicators were defined to evaluate the appropriateness of SPEP orders. Each indicator addressed a specific dimension in the request process and was associated with a predefined desirable target.

1. Indicator 1: The laboratory profile request is not clinically indicated [1].
  – Type: Structure indicator.
  – Description: Evaluates the presence of analytical profiles within the order entry system that include the SPEP test despite them not being specifically designed for the diagnosis or follow-up of monoclonal gammopathies.
  – Formula: Presence or absence of inappropriate laboratory profiles (qualitative value: Yes/No).
  – Desirable goal: Eliminating any laboratory profiles that routinely include SPEP in clinical settings other than monoclonal gammopathies.
2. Indicator 2: SPEP test orders without any suggestive laboratory findings [1], 9], 11].
  – Type: Process indicator.
  – Description: Proportion of SPEP orders with no evidence of suggestive laboratory findings (e.g. hypercalcemia, anemia or kidney failure).
  – Formula: Number of orders with no evidence of biochemical findings/Total orders.
  – Desirable target: <0.45.
  – Source of the target value: Local consensus aligned with SEMEDLAB/“*Choosing Wisely”* recommendations and monoclonal gammopathy epidemiology, with priority given to patients≥50 years.
3. Indicator 3: Orders in patients ≥50 years [1], [6], [7], [8, 10], 12].
  – Type: Process indicator.
  – Description: This indicator evaluates the proportion of SPEP orders for patients≥50 years, since this test is primarily useful in this population.
  – Formula: Orders in patients ≥50/Total orders.
  – Desirable target: >0.80.
  – Source of the target value: Local consensus aligned with SEMEDLAB/“*Choosing Wisely”* recommendations and monoclonal gammopathy epidemiology, with priority given to patients≥50 years.
4. Indicator 4: Activity per population [1].
  – Type: Activity indicator.
  – Description: Volume of SPEP reports in patients≥50 years per 1,000 population in the area (activity indicator adjusted to the target population).
  – Formula: (Determinations in patients ≥50 years/Population ≥50 years) × 1,000.
  – Desirable goal: Maintaining a stable trend or achieving a slight decrease post intervention [13].

### Implementation

– Firstly, the protocol was distributed among all Clinical Leads via the corporate e-mail.
– Then, the protocol was published on the corporate website.
– Next, the medical staff received training sessions on the protocol.

## Results

Between 2023 and the first quarter of 2025, a sustained decrease was observed in the volume of orders for patients <50 years (from 23.5 to 14.1 %), whereas orders for patients ≥50 years increased (from 76.5 to 85.9 %). In relation to Indicator 1, the desirable target was achieved following removal of the non-indicated orders detected in 2023. Indicator 2 (orders with no evidence of laboratory findings) worsened in 2024 (0.66) and partially improved in 2025 (0.53) even above the target value (<0.45). In 2025, Indicator 3 exceeded the target value (0.86). The activity per 1,000 population ≥50 years remained stable [6], [7], [8].

Orders for patients <50 years primarily decreased in Primary Care, Gastroenterology, Nephrology, and Pediatrics, whereas an increase was observed in the proportion of orders from the Unit of Rheumatology. These differences in ordering patterns suggest variability in clinical practice and diagnostic suspicion criteria, which should be addressed through specific training interventions [9].

**Figure 2:**
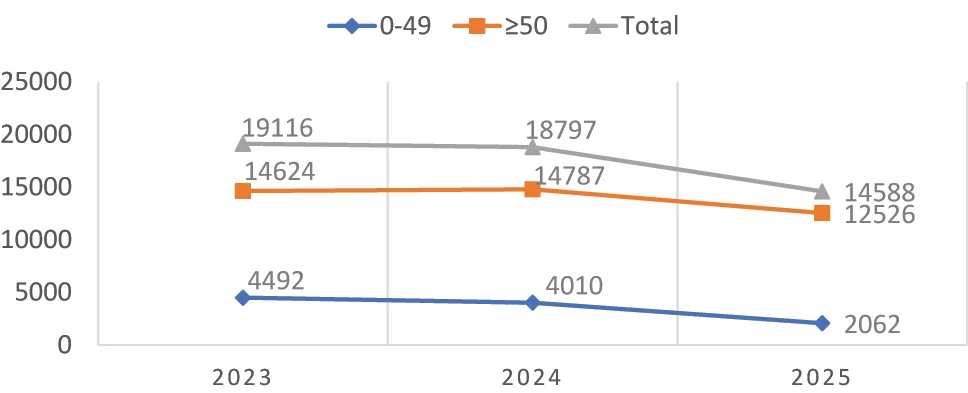
Evaluation of SPEP orders in valladolid oeste health district between January 1, 2023 and June 30, 2025. 2025 data were extrapolated from data for the first half of 2025.

Ordering patterns are detailed in Tables 1–3 and Figure 2.

**Table 1:** SPEPs performed in Valladolid Oeste Health District between January 1, 2023 and June 30, 2025.

Age	SPEPs n (%) 2023	SPEPs n (%) 2024	SPEPs n (%) first quarter of 2025
0–49	4,492 (23.5)	4,010 (21.33)	1,031 (14.13)
≥50	14,624 (76.5)	14,787 (78.67)	6,263 (85.87)
Total	19,116 (100)	18,797 (100)	7,294 (100)

The protocol automatically restricted the test in patients <40 years; a threshold 0–49/≥50 was established to analyze results due to its epidemiologic relevance and to allow comparison with the literature [1]. SPEP orders %: percentage with respect to the total SPEPs ordered.

**Table 2:** SPEPs performed in Valladolid Oeste Health District between January 1, 2023 and June 30, 2025. Others include: Allergology, Clinical Analysis, Pathology, Anesthesia and Resuscitation, Cardiology, General and Digestive Surgery, Plastic Surgery, Dermatology, Endocrinology, Geriatrics, Gynecology and Obstetrics, Intensive Care Medicine, Preventive Medicine, Microbiology and Parasitology, Pulmonology, Neurosurgery, Ophthalmology, Medical Oncology, Otolaryngology, Occupational Risk Prevention, Psychiatry, Radiology, Rehabilitation, Traumatology, Emergency Medicine, and Urology.

Service:	Total			<50 years		
	2023	2024	First semester of 2025	2023	2024	First quarter of 2025
Hematology	5,910	5,674	2,630	909	772	292
Primary care	3,618	3,645	1,090	1,162	1,006	150
Gastroenterology	2,684	2,422	506	824	655	128
Internal medicine	2,356	2,500	1,181	541	509	158
Nephrology	1748	1,191	519	296	194	35
Neurology	984	901	285	207	182	47
Rheumatology	586	1,206	587	138	312	120
Pediatry	111	78	12	109	72	8
Other	1,119	1,180	484	306	308	93

**Table 3:** Evaluation of indicators of appropriateness of SPEP test demand in valladolid oeste health District between January 1, 2023 and June 30, 2025. The 2025 data were extrapolated from data from the first half of 2025.

Indicator	Desirable target	R 2023	F 2023	R 2024	F 2024	R 2025	F 2025
Are there any test set orders unrelated to myeloma that include a SPEP test?	No	Yes	No	No	Yes	No	Yes
Ratio of orders with no laboratory findings	<0.45	0.45	No	0.66	No	0.53	No
Ratio of orders in patients ≥50 years	>0.80	0.77	No	0.79	No	0.86	Yes
Reported test results ≥50 years per 1,000 inhabitants	Stabilized or decreased moderately	55.26	Yes	54.99	Yes	46.12	Yes

R, result; F, favorable.

## Discussion

The results of this study demonstrate that the intervention protocol was effective in progressively improving SPEP ordering practices in the Valladolid Oeste Health District. Hence, the proportion of SPEP orders for patients <50 years decreased from 23.5 % in 2023 to 14.1 % during the first quarter of 2025. Such reduction aligns with AEBM-ML and SEQC-ML criteria, which do not recommend this test in patients younger than 50 in the absence of suggestive clinical evidence [1], [6], [7], [8, [10], [11], [12].

Additionally, the target value (>0.80) for Indicator 3 – which evaluates the percentage of orders for patients ≥50 years – was reached in 2025. This change shows that SPEP tests are requested for the appropriate population, in line with the epidemiological evidence that monoclonal gammopathies are most commonly diagnosed in patients older than 50 [6], [7], [8, [10], [11], [12].

In contrast, Indicator 2 (ratio of orders without suggestive laboratory findings) significantly worsened from 0.45 in 2023 to 0.66 in 2024, and decreased slightly to 0.53 in 2025 without reaching the target (<0.45). These findings suggest that, although the volume of orders has decreased, the SPEP test is still ordered without any supporting clinical or biochemical evidence. Otherwise said, there is still room for improvement that may be achieved by providing training to the medical staff and adopting more restrictive settings on test order systems. Age-based analysis revealed that orders not supported by laboratory findings were primarily observed within the 40–49 year age range. Therefore, efforts should be focused on improving the clinical selection of the younger subgroup. These results complement the results for Indicator 3 and underline the need to intensify medical staff training in SPEP ordering criteria for patients younger than 50 [8], 12].

The structure indicator demonstrated a positive change from the 15 inappropriate profiles detected in 2023 to zero. This structure intervention was essential to reduce the routine inclusion of SPEP orders in predefined profiles. This is consistent with the findings of recent studies that identify the elimination of non-indicated profiles as an effective approach to improve the appropriateness of diagnostic test orders [14].

In relation to Indicator 4, the activity per 1,000 population ≥50 years remained stable (from 55.26 to 54.99). The reduction achieved in 2024 (from 54.99 to 46.12) is the result of the intervention implemented to rationalize laboratory order practices and the elimination of non-indicated profiles. In 2025, a stabilization of this Indicator was observed, thereby suggesting a more selective use of SPEP.

The volume of orders for patients <50 years decreased substantially in Primary Care and the Internal Medicine Unit. In contrast, orders for this patient group increased significantly in the Unit of Rheumatology between 2023 and 2025. This variation could be due to changes in diagnostic practices or a higher awareness in this specialty of undiagnosed gammopathies, and therefore require qualitative analysis.

In summary, the protocol proved to be significantly effective in redirecting demand to appropriate age and clinical groups. However, there is still room for improvement in the biochemical support of test orders.

### Highlights

– SPEP is not a general screening test and should be ordered only for patients ≥50 years or in the presence of clinically supported suspicion [1], 3], [8], [9], [10].
– The protocol combined age-based restrictions, extended biochemical criteria, and training interventions. These measures were effective in controlling SPEP demand [3], [4], [5].
– The proportion of orders for patients ≥50 years increased (target achieved), and non-indicated profiles were removed from the order system.
– Orders lacking clinical indication persisted, especially in the 40–49 age group, which emerges as a primary target for improvement [11].
– Regular audits should be performed to further improve diagnostic yield [15].

## Conclusions

The appropriateness of SPEP orders in ASVAO improved with the implementation of the protocol, which involved focusing activity in patients ≥50 years and eliminating non-indicated laboratory profiles. PSEP orders with no supporting biochemical evidence remains to be high, which highlights the need to reinforce training and laboratory order filters. Regular monitoring of indicators will help consolidate improvements and detect deviations early [11], 15].

The reduction in SPEP orders has contributed to improve healthcare standards. The SPEP test is exclusively used for the diagnosis and clinical monitoring of monoclonal gammopathies. Furthermore, the reduction in the clinical workload for the lead clinician has enhanced the quality of the diagnostic process, allowing for a more efficient allocation of time and resources to cases where protein electrophoresis is clearly indicated.
